# Evaluation of Microscopic Observation Drug Susceptibility (MODS) and the string test for rapid diagnosis of pulmonary tuberculosis in HIV/AIDS patients in Bolivia

**DOI:** 10.1186/s12879-015-0966-0

**Published:** 2015-06-06

**Authors:** Meredith H. Lora, Melissa J. Reimer-McAtee, Robert H. Gilman, Daniel Lozano, Ruth Saravia, Monica Pajuelo, Caryn Bern, Rosario Castro, Magaly Espinoza, Maya Vallejo, Marco Solano, Roxana Challapa, Faustino Torrico

**Affiliations:** Department of Medicine, University of California in San Francisco, 505 Parnassus Ave, Rm 987, San Francisco, CA 94143-0119 USA; Department of Medicine, Tulane University School of Medicine, New Orleans, LA USA; Johns Hopkins Bloomberg School of Public Health, Baltimore, Maryland USA; Facultad de Medicina, Universidad Mayor de San Simón, Cochabamba, Bolivia; Colectivo de Estudios Aplicados y Desarrollo Salud y Medio Ambiente, Cochabamba, Bolivia; Universidad Peruana Cayetano Heredia, Lima, Peru; University of California in San Francisco, San Francisco, USA; La Escuela Técnica de Salud, Cochabamba, Bolivia

**Keywords:** Tuberculosis, HIV, AIDS, MODS, Bolivia, Resource-scarce, String test

## Abstract

**Background:**

Tuberculosis (TB) is the most common opportunistic infection and the leading cause of death in HIV-positive people worldwide. Diagnosing TB is difficult, and is more challenging in resource-scarce settings where culture-based diagnostic methods rely on poorly sensitive smear microscopy by Ziehl-Neelsen stain (ZN).

**Methods:**

We performed a cross-sectional study examining the diagnostic utility of Microscopic Observation Drug Susceptibility liquid culture (MODS) versus traditional Ziehl-Neelsen staining (ZN) and Lowenstein Jensen culture (LJ) of pulmonary tuberculosis (TB) and multidrug-resistant tuberculosis (MDRTB) in HIV-infected patients in Bolivia. For sputum scarce individuals we assessed the value of the string test and induced sputum for TB diagnosis. The presence of *Mycobacterium tuberculosis* (Mtb) in the sputum of 107 HIV-positive patients was evaluated by ZN, LJ, and MODS. Gastric secretion samples obtained by the string test were evaluated by MODS in 102 patients.

**Results:**

The TB-HIV co-infection rate of HIV patients with respiratory symptoms by sputum sample was 45 % (48/107); 46/48 (96 %) were positive by MODS, 38/48 (79 %) by LJ, and 30/48 (63 %) by ZN. The rate of MDRTB was 9 % (4/48). Median time to positive culture was 10 days by MODS versus 34 days by LJ (p < 0.0001). In smear-negative patients, MODS detected TB in 17/18 patients, compared to 11/18 by LJ (94.4 % vs 61.0 %, p = 0.03 %). In patients unable to produce a sputum sample without induction, the string test cultured by MODS yielded Mtb in of 9/11 (82 %) TB positive patients compared to 11/11 (100 %) with induced sputum. Of the 10 patients unable to produce a sputum sample, 4 were TB-positive by string test.

**Conclusion:**

MODS was faster and had a higher Mtb detection yield compared to LJ, with a greater difference in yield between the two in smear-negative patients. The string test is a valuable diagnostic technique for HIV sputum-scarce or sputum-absent patients, and should be considered as an alternative test to induced sputum to obtain sample for Mtb in resource-limited settings. Nine percent of our TB+ patients had MDRTB, which reinforces the need for rapid detection with direct drug susceptibility testing in HIV patients in Bolivia.

## Background

Tuberculosis (TB) is the most common opportunistic infection and the leading cause of death in HIV-positive people worldwide [1]. Diagnosing TB is difficult, and is more challenging in resource-scarce settings where those without timely culture-based diagnostic methods rely on poorly sensitive smear microscopy using Ziehl-Neelsen stain (ZN) [1–3].

Co-infection with HIV and TB affects the diagnosis of TB, as well as the morbidity, and mortality of patients in a multi-faceted manner. TB diagnosis in HIV is complicated by pauci-bacillary infection and atypical presentations of disease [4]. TB has been shown to increase HIV viral replication and may accelerate HIV progression [5] and increase mortality [6–8].

In the HIV population, smear microscopy detects only 35-61 % of culture-positive pulmonary TB infections [7, 9, 10], compared to 50-80 % in HIV-negative patients [3]_._ Given the increased incidence and mortality of smear-negative TB in HIV patients, culture is recommended [2, 3], but not widely used. When performed, culture improves detection of active TB; however, commonly used solid culture methods, including Lowenstein Jensen culture (LJ), may take 6–8 weeks, and lack concurrent antibiotic sensitivity data [3, 11, 12]. Ultra rapid test GeneXpert is currently recommended by the WHO as a first line TB test in the HIV population [13], but it is limited by lower sensitivity in smear negative patients and cost. At $10/cartridge and $17,000/machine, cost remains a barrier to widespread implementation, therefore alternative methods for rapid TB diagnosis in HIV patients are needed in resource limited areas [14–16].

### TB-HIV co-infection and Multidrug-resistant Tuberculosis (MDRTB) in Bolivia

Bolivia has the highest rate of active TB in South America, and one of the highest in the world, with an estimated prevalence of 196 in 100,000 [1]. Although only 11,421 total cases of HIV were reported in 2014, it was estimated that 15,000 persons were living with HIV in Bolivia, and 1000 deaths were attributed to AIDS [17]. Despite the prevalence of TB infection in Bolivia, WHO reported only 170 total patients with HIV-TB co-infection in 2013 [1].

Worldwide prevalence of MDRTB is increasing, and remains underdiagnosed [1]_._ It is estimated that only 7 % of the 500,000 people with MDRTB are recognized as MDR, which may lead to increased treatment failure and jeopardize the success of TB control efforts [1]. The WHO reported 1.2 % of newly diagnosed TB cases in Bolivia in 2013 were MDR [1]. In parts of the world where either MODS or GeneXpert are available, the diagnosis of either MDRTB or Rifampicin-resistant TB is made when the test returns positive for TB [13, 18, 19]. Currently, in Bolivia, however, diagnosis of MDRTB relies on LJ followed by further antimicrobial sensitivity testing, which can delay drug susceptibility results for weeks after initial diagnosis [20].

### Microscopic Observation Drug Susceptibility Culture (MODS)

The MODS assay is a low-cost, liquid culture used for the diagnosis of TB and direct drug susceptibility testing which uses commonly available laboratory materials in order to permit its application in resource-limited settings [18, 19]. MODS is based on the principles that Mtb grows faster in liquid than in solid medium and characteristic cording formation that is specific for Mtb is microscopically observed early in liquid medium [18]. The incorporation of Rifampicin and Isoniazid during the initial stage of the culture permits concurrent and direct drug-susceptibility testing, obviating the need for subculture sensitivity testing [18, 19]. Currently MODS costs $3 per test, and the recent development of a low-cost inverted microscope, automated plate readers, and a standardized TB MODS KIT will streamline the cultivation process, conserve labor hours preparing necessary reagents, and may further decrease costs [21, 22]. In a policy statement in 2011, the WHO approved MODS for drug susceptibility screening for MDRTB [23]_._

### Scarce sputum production in HIV patients

Obtaining an adequate sputum sample from patients with scarce sputum production is challenging in HIV-positive patients. Lack of productive cough in AIDS patients correlates poorly with the presence of pulmonary TB [24]. The most widely used option for sample obtainment is sputum induction. The string test, originally demonstrated for the retrieval of enteropathogens from the upper gastrointestinal tract [25, 26], has been shown to be a safe method of sample collection for the diagnosis of pulmonary TB in HIV patients [24].

Our study examines the diagnostic utility of Microscopic Observation Drug Susceptibility liquid culture (MODS) versus traditional Ziehl-Neelsen staining (ZN) and Lowenstein Jensen culture (LJ) of pulmonary tuberculosis (TB) and multidrug-resistant tuberculosis (MDRTB) in HIV-infected patients in Bolivia. For sputum scarce individuals we assessed the value of the string test and induced sputum for TB diagnosis.

## Methods

### Study patients and setting

This was a prospective and consecutive cross-sectional study conducted from February 2010 through August 2011 in Cochabamba, Bolivia, a city identified as an endemic TB region with an incidence of smear-positive pulmonary TB of 41.4 per 100,000 in 2011 [2].

All study participants were HIV-positive patients, confirmed by Western blot, with respiratory symptoms such as cough or dyspnea. Constitutional symptoms and risk factors for tuberculosis were noted on further evaluation but not assessed for study inclusion. Hospitalized subjects were evaluated upon admission to the infectious diseases ward of the Universidad San Simon, the public hospital in Cochabamba. Ambulatory subjects were evaluated upon presentation to the clinic with respiratory symptoms. Subjects were excluded if they were under 18 years of age, unwilling or unable to provide informed consent or actively receiving tuberculosis therapy for more than seven days. Study protocol and consent forms were approved by the institutional review boards of Universidad Peruana Cayetano Heredia, Asociación Benefica PRISMA, and Johns Hopkins Bloomberg School of Public Health.

### Sample collection

Participants were asked to submit three early-morning sputum samples and a gastric secretion sample by the string test. The first sample was divided into three aliquots to be processed by ZN, LJ, and MODS Two additional sputum samples were sent for ZN smear. Patients unable to produce adequate sputum (defined as minimum volume of 1 ml/culture sample of what macroscopically appeared to be sputum) were induced by a nebulizer with 3-5 % saline solution for up to three 5-min-intervals. Later in the study, all patients were asked to submit both a spontaneous sample and an induced sputum sample for comparison. Patients able to produce sputum spontaneously were considered “sputum productive.” Patients unable to produce a spontaneous sputum sample but with an adequate induced sputum sample were considered “sputum-scarce.” Patients unable to produce a sputum sample despite sputum induction were considered “sputum-absent.” Those willing to take part in the string test swallowed a gelatin capsule hand-packed with a 90 cm string after 8 hours of fasting. The trailing end of the string was attached to the patients’ cheek when swallowed. After one hour of intragastric time [27] the string was retrieved and the intragastric contents on the string were cultured by MODS as described below. If consented but unable to submit at least one sample (either sputum or string), they were excluded from study.

### Laboratory methods

String test samples were processed according to protocols described in previous publications^25^. Sputum and gastric samples were stored at 4 °C and were cultured as soon as possible after collection (Median 3.5 days, IQR 1–6). All specimens were decontaminated by the standard NALC-NaOH-Na citrate method [28]. MODS was performed as described in previous publications [18, 19] and cultures were examined every other day (excluding weekends) until day 30. LJ cultures were performed according to the protocol set by the National TB Program of Bolivia, and samples were inoculated at 37 °C and examined twice weekly until day 60. Rifampin and Isoniazid susceptibilities in this study were assessed through MODS alone.

### Statistical analysis

Data were analyzed using STATA 10, PRISM, and Excel software. Comparisons of categorical data were performed with either the Chi-squared test or Fisher’s exact test; comparisons of continuous data were performed with either *t*-test or Wilcoxon rank sum test. Comparisons of diagnostic yield between the different methods were assessed using McNemar’s Chi-square test or McNemar’s exact test.

As all patients had respiratory symptoms, a patient with a sputum *or* string test sample that was culture positive by Lowenstein Jensen or MODS was considered TB positive (“definite TB” according to 2012 NIH Consensus guidelines). As our study is evaluating MODS, with a known higher sensitivity than the reference test, this composite culture positivity was used as the reference standard to evaluate each test. As a result, our results reflect diagnostic yield of each detection method; we are unable to comment on the true sensitivity or specificity of each method. Standard anti-tuberculosis treatment was administered to patients with a positive result by any of the above diagnostic methods by the attending on service after communication of the positive results. Although this study was not initially designed to determine mortality, we did note if patients died by the time that his or her culture results were available. We defined mortality as death within 60 days of sample collection, the longest time period in which we would have contacted a patient with culture results.

## Results

### Samples obtained and patient characteristics

**Fig. 1:**
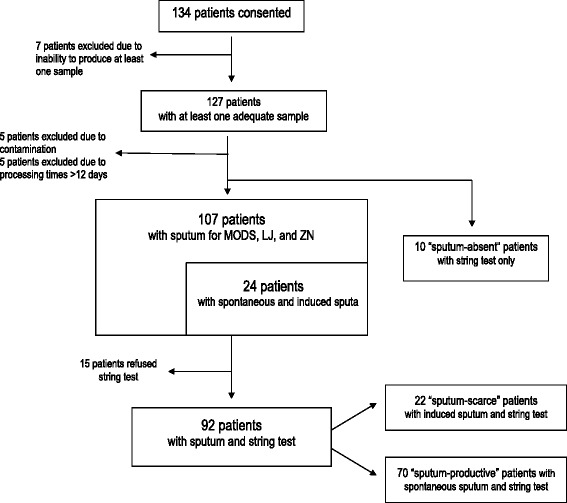
Recruitment of patients, samples obtained, and resulting groups of patients for sample analysis. 134 patients were enrolled in the study. 7 patients were unable to produce at least one sample (either string or sputum) and were excluded. 5 patients were excluded as their cultures were contaminated. 5 samples had long delays in processing by lab (>12 days); these patients were excluded. 107 subjects submitted a sputum sample sufficient for evaluation by ZN, LJ, and MODS that that were included in the analysis. Additionally, 24 of these patients submitted both induced and spontaneous sputum sample for comparison. Of the 107 patients who submitted an adequate sputum sample, 92 also submitted a string sample. 10 additional patients submitted string sample but did not submit sputum sample. Of these102 patients that submitted a string test sample, 70 patients (68.6 %) were able to produce sputum spontaneously (“sputum productive”). 22 patients (21.5 %) were only able to produce a sputum sample after induction (“sputum-scarce”). Ten patients (9.8 %) were unable to produce any sputum (“sputum-absent”). While we note the results of these 10 string samples, these ten patients were not included in our larger analysis of TB diagnostic methods as there was no sputum for comparison

Of the 134 patients enrolled in the study, 107 (79.8 %) subjects submitted sputa that were evaluated by ZN, LJ, and MODS. Additionally, 24 of these 107 patients (22.4 %) submitted both induced and spontaneous sputum sample for comparison. Of the 107 patients who submitted sputum, 92 (85.9 %) also submitted a string sample. 10 patients were unable to produce sputum but submitted a string test. Of those who submitted a string sample (n = 102), the patients were grouped into “sputum productive” (68.6 %), “sputum scarce” (21.5 %), or “sputum absent”(9.8 %), based on their degree of sputum production (See Fig.1).

The median age of the patients was 32 years (IQR 27–43). 101/107 patients (94.4 %) reported cough; median duration of cough prior to presentation was 20.5 days (IQR 7–60). 25/107 patients reported hemoptysis (23.3 %). 65/107 patients reported shortness of breath (60.7 %). 81/107 (75.7 %) of patients reported fever, 68/107 (63.5 %) of patients reported night sweats. 56/107 (52.3 %) reported both fevers and night sweats. The median CD4 count for all 107 patients was 98 cells/mm^3^; for hospitalized patients was 76.5; for ambulatory patients was 192 (Table 1). Only 28 of the 107 patients (26.1 %) were on ARVs at the time of sample collection. Of the TB+ patients, 12/48 (25.0 %) were on ARVs.

**Table 1:** Patient Characteristics: Median ages and CD4 counts, rates of TB positivity, and associated mortality of all TB+ patients that submitted sputum for comparison, stratified by hospitalized and ambulatory groups

	n	Median Age (IQR)	Median CD4 count (IQR)	TB+ (%)	Mortality of TB+
All patients	107	32 (27–43)	98 (33–249)	48/107 (44.9 %)	22/48 (45.8 %)
Hospitalized patients	76	32 (27–46)*	76.5 (29.0-162)**	35/76 (46.1 %)***	18/35 (51.4 %)****
Ambulatory patients	31	32 (28–42)*	192 (64–339)**	13/31 (41.9 %)***	4/13 (30.7 %)****

107 patients were included in analysis. Median age of all patients as well as those hospitalized vs ambulatory was 32. Median CD4 count for all patients was 98. Median CD4 count of hospitalized patients was 76 and median CD4 for ambulatory patients was 192. Rates of TB positivity (TB+), defined as a patient with a sputum or gastric string sample that was culture positive by MODS or Lowenstein Jensen, was 44.9 % in all patients, 46.1 % in hospitalized patients, and 41.9 % in ambulatory patients. Of those who were TB+, the mortality rate overall was 45.8 %. The mortality rate for hospitalized TB+ patients was 51.4 % and for ambulatory TB+ patients, it was 30.7 %

*n* number of patients in each group. Medians are displayed in year. *IQR* Interquartile range

*The difference between the median age of hospitalized versus ambulatory patients was not statistically significant (p = 0.87)

**The difference between the median CD4 count of hospitalized versus ambulatory (76.5 vs 192) was statistically significant (p = 0.01)

**The difference between the rates of TB of hospitalized vs ambulatory patients (46.1 % vs 41.9 %) was not statistically significant (p = 0.70)

***The difference between the mortality of the TB+ hospitalized patients vs mortality of TB+ ambulatory patients (51.4 % vs 30.7 %) was not statistically significant (p = 0.08)

### Rates of TB and Associated Mortality of TB+ pts

Of the 107 patients, 44.9 % (48/107) were Mtb culture positive by MODS or LJ culture of the sputum or string test sample and were considered TB+. The rates of TB in hospitalized patients (n = 35, 46.1 %) versus ambulatory (n = 13, 41.9 %) were similar. The mortality rate of all TB+ patients was 45.8 % (22/48) (see Table 1). Of those TB+ sensitive to both rifampin and isoniazid, mortality was 35.0 % (14/40); of those TB+ with resistance to one or more anti-TB drugs, mortality was 75 % (6/8); p = 0.08.

Of the 22 TB+ patients that died during the study, the median CD4 count was 59 (IQR 29–132.5), compared to 174 (IQR 52–257) in surviving TB+ patients (n = 26). Between these two groups, their median duration of symptoms prior to presentation was 21 days in deceased patients (IQR 4.75-97.5) versus 30 days in surviving patients (IQR 9.25-97.5). The rates of antiretroviral therapy at the time of enrollment in these two groups were similar (5/22 of deceased patients, 6/26 of surviving patients, p = 0.62).

### Mtb Detection and Time to Positivity

MODS detected Mtb in 46/48 TB+ patients with a diagnostic yield of 95.8 % (95 % CI, 85.7-99.5), and detected Mtb in significantly more patients than LJ (38/48, 79.2 %, p = 0.022) and ZN (30/48, 62.5 %, p < 0.001). In the ambulatory patients, MODS detected Mtb in 12/13 TB+ patients (92.3 %) versus LJ in 7/13 (53.8 %), p = 0.06, and the detection yield of LJ was similar to that of ZN (53.8 % vs 61.5 %, p = 1.00). See Table 2.

**Table 2:** Comparison of diagnostic methods for the detection of Mtb in hospitalized versus ambulatory and in smear-positive versus smear-negative patients

A.	All patients		Hospitalized patients		Ambulatory patients	
	TB+ (n = 48)	Diagnostic yield (95 % CI)	TB+ (n = 35)	Diagnostic yield (95 % CI)	TB+ (n = 13)	Diagnostic yield (95 % CI)
MODS	46	95.8 % (85.7-99.5)	34	97.1 % (85.1-99.9)	12	92.3 % (64.0-99.8)
LJ	38	79.2 % (65.0-89.5)	31	88.6 % (73.3-96.8)	7	53.8 % (25.1-80.8)
ZN	30	62.5 % (47.3-76.0)	22	62.9 % (44.9-78.5)	8	61.5 % (31.6-86.1)
B.	All patients		Smear-positive		Smear-negative	
	TB+ (n = 48)	Diagnostic yield (95 % CI)	TB+ (n = 30)	Diagnostic yield (95 % CI)	TB+ (n = 18)	Diagnostic yield (95 % CI)
MODS	46	95.8 % (85.7-99.5)	29	96.6 % (82.8-99.9)	17	94.4 % (72.7-99.9)
LJ	38	79.2 % (65.0-89.5)	27	90.0 % (73.4-97.9)	11	61.0 % (35.7-82.7)

TB+ was defined as any patient with sputum or gastric sample that yielded a positive culture result by LJ or MODS. As the reference standard is a composite from all tests that are being evaluated, we can only comment on diagnostic yield rather than sensitivity and specificity. Panel A: Comparison of diagnostic yield of MODS versus LJ and ZN in all patients, hospitalized patients, and ambulatory patients who were TB positive. Panel B: Subgroup comparison of the diagnostic yield of MODS versus LJ in culture positive patients, sub-grouped into all patients, smear positive patients, and smear negative patients

*n* number of patients in each group; *CI* confidence interval; *TB +* samples tested positive for Mtb by each respective method (MODS, LJ, or ZN)

Thirty-seven percent (18/48) of TB+ patients were smear-negative. In the smear-negative group, MODS detected TB in 17/18 patients, significantly more detected than the 11/18 by LJ (94.4 % vs 61.0 %, p = 0.03 %). In the smear-positive group (30/48) the difference was not significant (MODS 96.6 % and LJ 90.0 %, p = 0.63). See Table 2.

**Fig. 2:**
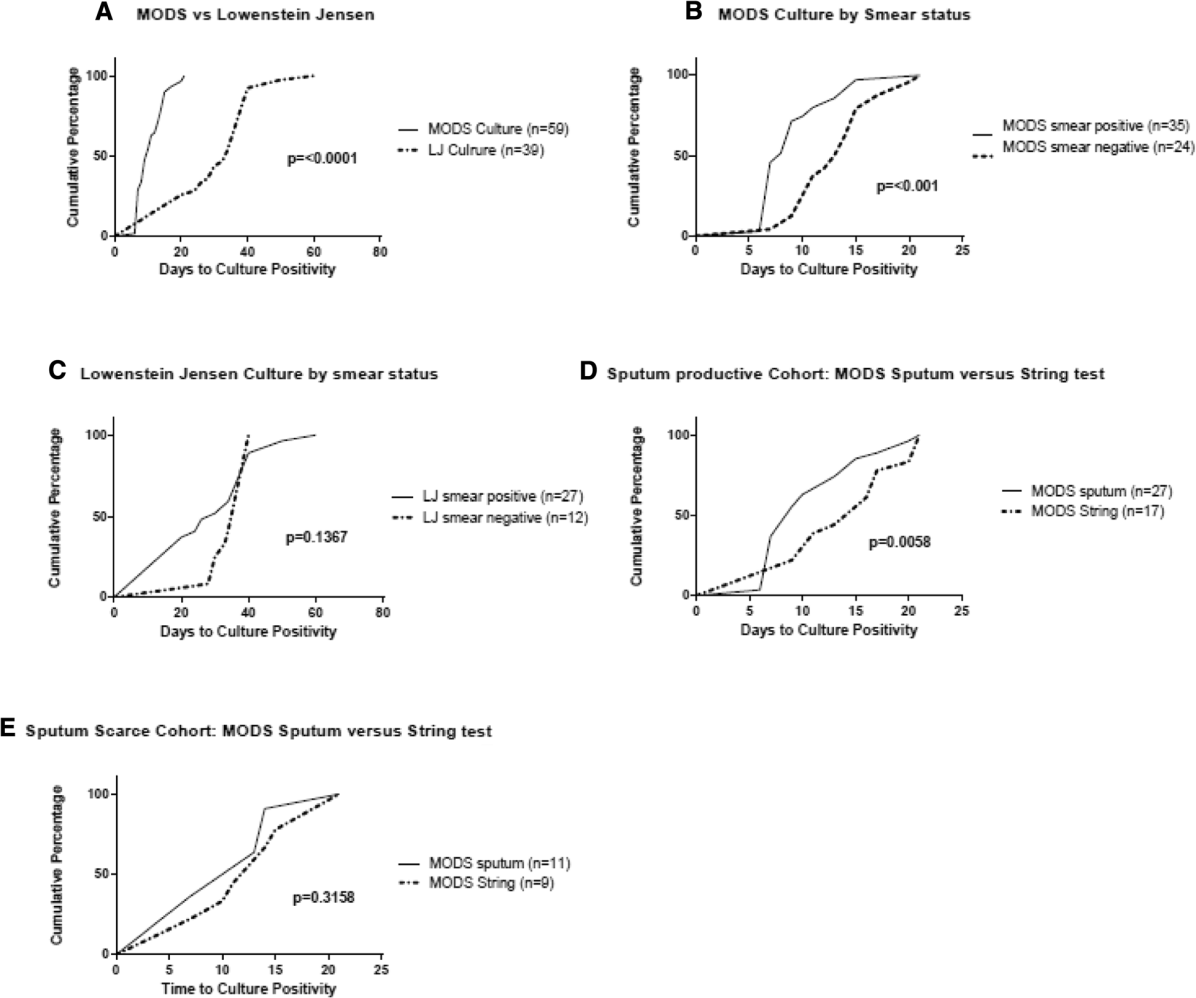
Cumulative percentages of the time to culture positivity for culture-positive samples according to culture method (**a**), according to smear status of sputa (**b** and **c**), and according to sample type in sputum-productive and sputum-scarce patients (**d** and **e**). A. Median time to sputum culture positivity cultured by MODS vs LJ (10 days vs 34 days, p <0.0001). **b**. Within MODS culture of sputa, effect of smear-positive vs smear-negative on time to culture positivity (median 8 vs 13 days, p <0.001). **c**. Within LJ of sputa, effect of smear-positive vs smear-negative on time to culture positivity (median 30 vs 40 days, p = 0.1367) **d**. Within the sputum-productive cohort, time to culture positivity of MODS sputum vs MODS string test (median 9 vs 13 days, p = 0.0058). **e** Within the sputum-scarce cohort, time to culture positivity of MODS sputum vs MODS string test (median 11 vs 14 days, p = 0.3159). String samples were not neutralized *prior to* storage and this may have affected both diagnostic yield and time to positivity. Median times to processing with interquartile ranges for each group of samples are below: All MODS Sputum Samples: Median 3 days (IQR 1–5). MODS Smear-positive Sputum Samples: Median 4 days (IQR 2–5). MODS Smear-negative Sputum samples: Median 2 days (IQR 2–4.75). LJ Sputum samples: Median 5 days (IQR 2–6). All MODS String Samples: Median 3 days (IQR 1–5). MODS Sputum-Productive Sputum Samples: Median 3 days (IQR 2–5). MODS Sputum-Productive String Samples (Median 3 days (IQR 2–5). MODS Sputum-Scarce Sputum Samples: Median 4 days (IQR 2–6). MODS Sputum-Scarce String Samples: Median 4 days (IQR 2–6)

The median time to sputum culture positivity was significantly shorter for MODS than for LJ (10 and 34 days, p < 0.0001). Within MODS, smear status significantly affected time to positivity (median 8 versus 13 days in smear-positive and negative samples, p < 0.0001). This difference was not seen in LJ by smear status. See Fig. 2.

### The string test: performance in sputum-productive, sputum-scarce and sputum-absent patients

Sputum-scarce patients had a lower median CD4 count than sputum productive patients (55 versus 122; p = 0.021). 3 of 22 (13.6 %) sputum scarce patients were on ARVs at the time of enrollment compared to 21 of 90 (23.3 %) of sputum productive patients. The rate of TB+ in the sputum-scarce group was 50 % (11/22), compared to 40 % (28/70) in the sputum-productive patients (p = 0.41). Of those TB+, the sputum-scarce group had a higher mortality rate (8/11, 72.7 %) than the sputum-productive group (10/28 35.7 %), p = 0.041. See Table 3.

**Table 3:** String Test sub-analysis: Patient characteristics and comparison of diagnostic methods in sputum productive, sputum scarce and sputum absent patient subgroups

Patient Characteristics (n = 102)	Sputum-productive (n = 70)		Sputum-scarce (n = 22)		Sputum-absent (n = 10)	
Median age (years)	32		30.5		36	
CD4 count	122 (IQR 45–271)		55 (IQR 23–86)		221 (IQR 133–486)	
Rate of TB+	28/70 (40 %)		11/22 (50 %)		4/10 (40 %)	
Mortality of TB+	10/28 (35.7 %)		8/11 (72.7 %)		0/4 (0 %)	
Test	+Mtb (n = 28)	Diagnostic yield (95 % CI)	+Mtb (n = 11)	Diagnostic yield (95 % CI)	+Mtb (n = 4)	Diagnostic yield
String test MODS	18	64.2 % (44.7-81.4)	9	81.8 % (48.9-97.7)	4	^a^
Sputum MODS	27	96.4 % (81.7-99.9)	11	100 % (71.5-100)	^a^	^a^
Sputum LJ	21	75.0 % (55.1-89.3)	10	90.9 % (58.7-99.8)	^a^	^a^
Sputum ZN	17	60.7 % (40.6-78.5)	7	63.6 % (30.8-89.1)	^a^	^a^

TB positive was defined as any patient with sputum or gastric sample with a positive culture result from sputum or string test by LJ or MODS. This composite positivity was used as the reference standard to determine diagnostic yield. The patients that submitted a string test (n = 102) were divided into three groups based on degree of sputum productivity. Sputum-productive patients (n = 70, 68.6 %) were able to produce spontaneous sputum. Sputum-scarce patients (n = 22, 21.5 %) required sputum induction to produce sample. Sputum-absent patients (n = 10, 9.8 %) were unable to produce sputum despite induction and only submitted a string sample. Of the sputum absent patients, 4 patients were positive for Mtb by string test, but there are no samples for comparison since they were unable to produce sputum. The string test samples were not neutralized prior to culture and this may have affected diagnostic yield

*n* number of patients in group; *IQR* interquartile range; *CI* confidence interval

^a^ We are unable to provide these numbers as sputum absent patients did not submit sputum for comparison

Cultures of string test sample and sputum were compared by MODS. In the sputum-productive group (n = 70), of the 28 TB+ patients, culture of the string test yielded Mtb in 18/28 (64.2 %) patients compared to 27/28 (96.4 %) with sputum (p = 0.012). In the sputum-scarce group (n = 22), of the 11 TB+ patients, the string test yielded Mtb in 9/11 (81.8 %) patients compared to 11/11 (100 %) with induced sputum (p = 0.50). Ten patients with a dry cough were “sputum absent”, and were only able to submit a string sample; culture of the string test yielded Mtb in 4/10 of these patients. See Table 3.

In the sputum-productive group, time to positivity for sputum cultured by MODS was significantly faster than string test cultured by MODS (median 9 vs 15 days, p = 0.006). In the sputum-scarce group, time to positivity of induced sputum was similar to that of string test (median 11 vs 14 days, p = 0.31). The string test samples were not neutralized until just prior to culture, and this along with the processing delay may have negatively affected both diagnostic yield and time to positivity. See Fig. 2.

### Detection of Mtb in induced sputum versus spontaneous sputum using MODS culture

24 patients submitted both spontaneous and induced sputum. In this group, 14 patients were TB positive by at least one of the samples obtained. Of these 14 patients, MODS detected TB in 92.8 % (13/14) of the induced sputum samples vs 78.6 % (11/14) of the spontaneous sputum samples (p = 0.63). The median time to positivity for induced versus spontaneous sputum was not significantly different (11 vs 9 days, p = 0.30). See Fig. 2.

### Rates of MDRTB in TB-HIV co-infection

The rate of both Rifampin and Isoniazid resistance (MDRTB) in our study was 9.0 % (4/46). Rates of resistance to Rifampin alone was 4.0 % (2/46) and to Isoniazid alone was 4.0 % (2/46), thus a total of 17.0 % (8/46) of patients were resistant to *at least one* of these two medications. Resistance is determined concurrently with initial positive MODS culture, so time to resistance parallels that of time to positivity as described above.

## Discussion

The data from our study stresses the need for a rapid TB diagnostic test in HIV patients in Bolivia. The rate of TB in HIV patients with respiratory symptoms was very high (45 %), and almost half (46 %) of these patients died within 60 days. Furthermore, the rate of TB-HIV co-infection in *our study alone* over a 1.5 year period (n = 48) does not correlate well with the rate of HIV-TB co-infection in all of Bolivia reported in 2013 (n = 170) [1]; this data raises concern that this is a problem of greater magnitude than is currently appreciated. Even more alarming is the prevalence of MDRTB in our population of HIV patients (9 %), which is much higher than the reported MDRTB rates in Bolivia; and that 75 % of those with resistance to one or more drugs died during the duration of our study. A similar study by Kawai et al. in Peru showed that MDRTB-HIV co-infection had a mortality rate above 50 % within two months and was associated with a high rate of continued infectiousness [29]. Current methods of resistance testing in Bolivia can take weeks beyond positive solid culture results, risking significant delays of appropriate treatment. These data strongly reinforce the need for the implementation of a rapid TB diagnostic method with direct drug susceptibility testing in the HIV population in Bolivia. Additionally, the discrepancy between the rates of resistance found in our study compared to previous reports, calls for a dedicated study of antibiotic resistance in Bolivia in HIV patients. Given the increased rates of MDRTB in HIV, the risk of nosocomial spread, and the high mortality associated with this co-infection, if these rates of MDRTB are confirmed in future studies, MDRTB in Bolivia could quickly become a greater threat to public health.

Our study demonstrates that MODS is a valuable method for rapid diagnosis of TB in the HIV population. In this study, the detection yield of MODS was superior to LJ. Additionally, consistent with prior studies based in centralized MODS laboratories, Mtb was observed in MODS in less than one-third of the time of growth in LJ [18, 19, 23]. MODS’ increased diagnostic yield was most profound in the smear-negative HIV patients, a vulnerable group at highest risk for missed or delayed diagnosis. Although smear-negative sputum samples cultured by MODS grew later than MODS smear-positive samples, smear-negative samples by MODS still grew significantly faster than those by LJ.

Our results emphasize the utility of MODS in the ambulatory population. MODS detected almost 30 % more disease than LJ in this population, and LJ did not identify any more disease than ZN alone, supporting a use for MODS in patients with milder presentations.

As this study highlights the need for widespread implementation of a rapid, sensitive TB diagnostic method in HIV patients with direct drug susceptibility testing, it is important to mention the role of GeneXpert as compared to MODS. GeneXpert would also be a welcome alternative to the current standard of care in Bolivia for HIV patients. However, at the current time, without a committed long-term external funding source, its cost ($10/cartridge and $17,000/machine) limits widespread implementation in resource-limited settings like Bolivia [13]. MODS ($3/test) remains a more cost-effective alternative in these settings. Additionally, given MODS performance in smear negative patients as demonstrated in this study and others, and GeneXpert limitations in this domain [14, 15], we would also recommend MODS as a low cost alternative for GeneXpert for smear negative patients.

Our study provides data about sputum scarce HIV patients with respiratory symptoms in a TB endemic area. Despite minimal sputum production, the prevalence of TB in this population trended towards *higher* than the sputum-productive subjects and the 60 day mortality rate of those TB+ was greater in the sputum-scarce group than the sputum productive patients.

Additionally, we examine the value of the string test as a sample obtainment method for TB in sputum scarce or absent patients. In our sputum-scarce group, the string test detected over 80 % of TB cases, and was not significantly different than the number of TB cases by induced sputum. Importantly, in the 10 patients that could not produce a sputum sample despite induction, the string test detected 4 cases of TB that would have otherwise been missed; thus 40 % of patients that could not produce a sputum sample were diagnosed and received treatment because of the string test. Our diagnostic yield is lower than in previous studies [24], this may be due to lack of neutralization before storage and delayed processing of samples. Despite this, the string test provided an inexpensive, minimally invasive alternative for diagnosis of TB in sputum-scarce and sputum-absent patients without an associated biohazard risk. Given the above data, we suggest that the string test be considered as an alternate sample obtainment method used in conjunction with MODS in HIV patients with little to no sputum production.

Of the patients with both induced and spontaneous sputa (n = 24), although the difference of the results between the two groups were not statistically significant, two patients that were negative for TB by spontaneous sputum were TB+ by induced sputum. Although induction of sputum in the diagnosis of TB is historically more common in the pediatric population [30, 31], more recent studies evaluating the utility in adults in HIV-endemic regions have come to differing conclusions [32–35]. Small studies have promoted the use of induction of sputum by demonstrating an increased diagnostic yield when compared to spontaneous sputum [32–34]; however, a randomized controlled trial of 418 patients in South Africa did not show a significant difference between the results of healthcare worker-instructed versus induced sputum [35]. Furthermore, induction of sputum requires additional equipment, poses a biohazard risk, and is an increased cost to hospitals (US$2.14 vs $7.88) [35].

Additionally, our study is the first *in Bolivia* to demonstrate that MODS is superior to the current local standards of tuberculosis diagnosis. We showed that a laboratory with minimal prior experience with MODS is able to produce results similar to that of centralized MODS laboratories, as in Peru, where MODS is now the standard of care [23]._._ Furthermore, the implementation of MODS can be more simplified and affordable in the future, with the recent development of MODS kits and availability of inexpensive inverted microscopes [21–23].

Limitations of our study include our increased time to positivity compared to prior studies in Peru [18, 19]. This increase may have been influenced by delay in processing times of the samples, and the fact that samples were checked less often (due to personnel availability), rather than every day. Although most samples were cultured within 4 days of collection (Median 3.5, IQR 1–6), some were stored for up to 11 days, which may have affected growth of culture. The diagnostic yield of the string test in this study is lower than previous studies [24]; it is important to note our lack of neutralization of the sample before storage as well as delays in processing samples may have decreased string test yield and is a limitation of our study.

Our imperfect reference standard was an additional limitation to optimally evaluate the diagnostic performance of MODS. The performance of MODS would have been more clearly defined if our standard included other sensitive diagnostic methods such as MGIT or GeneXpert rather than a composite of all of the tests being evaluated. This was due to logistical limitations and financial constraints. Although the comparison of MODS to the standard solid culture media for diagnosis of TB in Bolivia provides important data for the community tested, the incorporation of GeneXpert in our study would have been a valuable comparison, had it been a possibility.

Additionally, there is a gap in our data regarding the mortality of the HIV patients that were negative for TB. Regrettably we did not record the mortality data for all patients without TB during the time of the study, thus we are not able to make a comparison in mortality between the TB-positive and TB-negative patients.

In many parts of the world, HIV is no longer a death sentence, but has become a manageable chronic disease due to increased detection and wider availability of effective treatments. The currently undertreated and highly lethal HIV-TB co-infection is a barrier to this becoming a reality in many TB-endemic areas such as Bolivia. Implementation of MODS, or if economically feasible, another rapid diagnostic test such as GeneXpert, should become the standard for diagnosis of TB in HIV populations in resource-scarce settings. This would empower clinicians to treat patients with effective antimicrobials at earlier time points, which would lead to significant effects on both patient mortality and public health through decreased transmission of this deadly disease.

## Conclusions

The high prevalence and mortality of TB-HIV co-infection found in our study and others highlights the need for a more rapid diagnostic test for TB that includes direct drug susceptibility testing in HIV patients in Bolivia and in similar resource-limited settings. MODS culture performs well in this setting, and should be considered as a low cost alternative in HIV patients. In addition, a more effective approach to symptomatic HIV patients with little or no sputum production is needed; the string test could be a valuable diagnostic tool for this subset of patients.

## Abbreviations

TB: Tuberculosis
ZN: Ziehl-Neelsen stain
MDRTB: Multidrug-resistant tuberculosis
MODS: Microscopic Observation Drug Susceptibility liquid culture
LJ: Lowenstein Jensen culture
Mtb: *Mycobacterium tuberculosis*
HIV: Human Immunodeficiency Virus
AIDS: Acquired Immunodeficiency Syndrome
